# The Impact of Deforestation, Urbanization, and Changing Land Use Patterns on the Ecology of Mosquito and Tick-Borne Diseases in Central America

**DOI:** 10.3390/insects13010020

**Published:** 2021-12-23

**Authors:** Diana I. Ortiz, Marta Piche-Ovares, Luis M. Romero-Vega, Joseph Wagman, Adriana Troyo

**Affiliations:** 1 Biology Program, Westminster College, New Wilmington, PA 16172, USA; 2 Laboratorio de Virología, Centro de Investigación en Enfermedades Tropicales (CIET), Universidad de Costa Rica, San José 11501, Costa Rica; maria.piche.ovares@una.cr; 3 Departamento de Virología, Escuela de Medicina Veterinaria, Universidad Nacional, Heredia 40104, Costa Rica; 4 Departamento de Patología, Escuela de Medicina Veterinaria, Universidad Nacional, Heredia 40104, Costa Rica; LUIS.ROMEROVEGA@ucr.ac.cr; 5 Laboratorio de Investigación en Vectores (LIVe), Centro de Investigación en Enfermedades Tropicales (CIET), Universidad de Costa Rica, San José 11501, Costa Rica; adriana.troyo@ucr.ac.cr; 6 Malaria and Neglected Tropical Diseases Program, Center for Malaria Control and Elimination, PATH, Washington, DC 20001, USA; jwagman@path.org; 7 Departamento de Parasitología, Facultad de Microbiología, Universidad de Costa Rica, San José 11501, Costa Rica

**Keywords:** arbovirus, malaria, deforestation, urbanization, Rickettsiales, *Culicidae*, *Ixodidae*, Central America

## Abstract

**Simple Summary:**

Central America is a region that possesses distinct ecological and socioeconomic characteristics, making it increasingly vulnerable to vector-borne diseases. The emergence and resurgence of these diseases has been linked to environmental changes driven by human activities, particularly land use changes associated with deforestation, forest degradation, and urbanization. However, the effects of these environmental modifications on the transmission dynamics and the increase of infection risks are not well understood in Central America where information is limited and scattered. In this article, we review and analyze the current knowledge and potential impacts of deforestation and urbanization on the risk and transmission dynamics of the most relevant mosquito-borne and tick-borne diseases in Central America. Disease events, such as the recent Zika and dengue epidemics, and the uneven progress towards regional malaria elimination highlight the need to increase awareness regarding the complex ecological interactions and environmental changes taking place in this region and how this information could be used to improve prevention and control strategies.

**Abstract:**

Central America is a unique geographical region that connects North and South America, enclosed by the Caribbean Sea to the East, and the Pacific Ocean to the West. This region, encompassing Belize, Costa Rica, Guatemala, El Salvador, Honduras, Panama, and Nicaragua, is highly vulnerable to the emergence or resurgence of mosquito-borne and tick-borne diseases due to a combination of key ecological and socioeconomic determinants acting together, often in a synergistic fashion. Of particular interest are the effects of land use changes, such as deforestation-driven urbanization and forest degradation, on the incidence and prevalence of these diseases, which are not well understood. In recent years, parts of Central America have experienced social and economic improvements; however, the region still faces major challenges in developing effective strategies and significant investments in public health infrastructure to prevent and control these diseases. In this article, we review the current knowledge and potential impacts of deforestation, urbanization, and other land use changes on mosquito-borne and tick-borne disease transmission in Central America and how these anthropogenic drivers could affect the risk for disease emergence and resurgence in the region. These issues are addressed in the context of other interconnected environmental and social challenges.

## 1. Introduction

Vector-borne diseases (VBDs) remain an important public health problem worldwide, particularly in tropical and subtropical regions, and they are becoming more prevalent in recent years. Arthropod vectors are associated with the transmission of some of the most significant infectious diseases affecting both animals and humans [1,2,3]. The global burden of VBDs is significant, accounting for more than 17% of infectious diseases in humans with more than three billion people currently inhabiting endemic areas and at risk of exposure to these pathogens [4]. Most people affected by these diseases live in developing countries under conditions that favor a greater burden of disease, especially in poor and marginalized populations, including rural inhabitants, Indigenous populations, women living in poverty, the elderly, and children [5,6]. Together, these diseases produce significant mortality and morbidity, causing millions of deaths every year, long-term disabilities, and life-long sequelae [5,7,8,9].

Emerging and resurging diseases are defined as recently evolved or newly discovered pathogens that have demonstrated increased incidence in host populations in the past 20 years or poses a future threat. These pathogens are characterized by their expanding geographic spread, increasing public health impact, changes in their clinical presentation, or novel infection occurrence in humans [10,11]. Some of these pathogens are also characterized by their resurgence after long periods of decline in infection incidence. About 60% of these diseases are zoonotic in origin and, to various degrees, dependent on animal reservoirs for survival and maintenance [10,11,12,13,14]. In many countries, the incidence of these diseases has declined to low levels due mostly to effective prevention and control programs; however, some were never satisfactorily controlled throughout their endemic regions. Many are currently increasing in incidence and spreading beyond their previously known geographical ranges. Some of them have reappeared only in limited regions while others have become major global problems [9,12,15].

Most emerging zoonotic VBDs are transmitted by ticks (Family *Ixodidae*) and mosquitoes (*Culicidae*) and caused by RNA viruses (Families *Flaviviridae*, *Bunyaviridae*, and *Togaviridae*) and Rickettsiaceae bacteria [12,16]. The vectorial capacity of mosquitoes and ticks is enhanced by their high environmental adaptability, which includes high reproductive outputs under suitable conditions and high capacity to invade ecologically disturbed environments, especially peridomestic habitats where human and domesticated animal hosts are readily available [16,17,18]. In terms of human morbidity and mortality, malaria, dengue, Rickettsial fevers, and Lyme disease are some of the most important of these resurgent infections [2,15,16,19].

The relationship between arthropod vectors and the pathogens they transmit is particularly sensitive to anthropogenically driven global changes. Factors behind the dramatic emergence and resurgence of VBDs are complex, vary geographically and temporarily, and often have an additive effect on disease ecology and epidemiology [1,2,12,16,19]. Currently recognized anthropogenic drivers of VBD emergence and resurgence include demographic changes (e.g., global population movements and growth, unplanned and uncontrolled urbanization), socioeconomic changes (e.g., modern transportation and commerce, human encroachment on natural disease foci), illegal activities (e.g., illegal logging and cattle ranching, illegal drug trafficking), accelerated exploitation of natural resources (e.g., changes in land use, forest degradation, reduction in biodiversity, agricultural practices), changes in host susceptibility and pathogen adaptation (e.g., increased movement of humans and animals, pathogen genetic variability), degradation of public health infrastructure (e.g., lack of effective vector control, disease surveillance, and prevention programs), and climate change (e.g., changes in regional temperature and rainfall patterns lead to alterations in vector dynamics) [1,2,12,16,19,20,21,22]. 

There is increasing evidence that anthropogenic land use changes can directly and indirectly influence vector-borne pathogen transmission dynamics [10,23]. Although land use changes, such as urbanization as a result of forest degradation and deforestation, have been associated with increased disease transmission, their direction, extent, specific mechanisms, and persistence are not clearly understood. Often the effects of these factors on VBD emergence and resurgence are interdependent, synergistic, and difficult to study.

Central America is the region that links North and South America comprising most of the narrow isthmus that separates the Pacific Ocean from the Caribbean Sea. It consists of the countries of Belize, Costa Rica, Guatemala, El Salvador, Honduras, Panama, and Nicaragua, and is inhabited by about 44.5 million people. Generally, this region is characterized by a diversity of ecosystems, physical geographies, sociocultural and socioeconomic structures, and public health profiles [24]. Over the past decades, this region has sustained dramatic land use changes, including cattle ranching, large-scale commercial plantations, illegal airstrips, mining, road construction, tourism infrastructure, illegal timber extraction, and housing construction. These land use changes have significantly accelerated the rates of deforestation and urbanization in the region [25,26,27,28,29,30]. Despite the high prevalence of several VBDs in Central America, the effects of anthropogenic-driven deforestation and urbanization on the transmission dynamics of these diseases are not well understood. The presence and interactions of numerous physical and socioeconomic factors in the region amplifies its vulnerability to the emergence and resurgence of several VBDs [6].

The purpose of this descriptive review is to profile the potential impact of deforestation, forest degradation, and urbanization on mosquito-borne and tick-borne disease transmission dynamics in Central America and how these anthropogenic drivers could affect risk for disease emergence and resurgence in the region. This review article focuses specifically on mosquito-borne and tick-borne pathogens currently causing high disease incidence and prevalence in humans or that have a high potential for emergence or resurgence in the region. To provide a proper context to these issues, our review also includes a brief history on deforestation and urbanization in Central America and the mosquito-borne and tick-borne diseases that have been recorded in the region. These diseases have received less attention than other VBDs in Central America, such as leishmaniasis and Chagas disease, which have been widely studied in the region and have been featured in comprehensive literature reviews [31,32,33,34,35]. This review assesses direct and indirect evidence and identifies knowledge gaps that could stimulate future research initiatives in the region. Understanding the causes and potential impacts of deforestation and urbanization on VBD transmission in Central America is essential to the improvement of current disease prevention and control approaches.

## 2. Deforestation in Central America

The Central American region is about 2200 km in length, northwest to southeast, and 600 km wide at its broadest point covering a total area of more than 550,000 square kilometers. The topography and vegetation in this region are defined by several mountain ranges that stretch extensive parts of the region’s length. Between these mountain ranges lie fertile valleys where most of the populations reside and where most of the agricultural activity occurs, such as raising livestock and the cultivation of coffee, beans, tobacco, and other crops [24,25]. Central America is also part of the Mesoamerican Biodiversity Corridor (MBC), a patchwork of diverse and protected biomes, established in 1997, that connects North and South America. It represents the world’s third largest biodiversity hotspot containing about 7–10% of the world’s known species [36]. Although Central American forests currently cover about 200,000 square kilometers, they were once much more extensive. About 12% of the MBC is protected land in the form of ecoregions and nature reserves [36,37]. Mesoamerican forests are highly susceptible to destruction and damage and have one of the highest rates of ecological degradation in the world [38]. However, the history and drivers of deforestation have taken different forms depending on the country and region [22,25,26].

In the 1960s and 1970s, Central America underwent the highest rate of deforestation in the world with an increasing number of settlers clearing the land for cattle ranching (the “hamburger connection”) and commercialization activities [25,39,40]. Over the last 30 years, Central America has experienced a rising demand for food and energy driving national authorities to exploit natural resources for energy generation and agricultural production. Numerous drivers of deforestation and forest degradation have significantly accelerated the pace of net forest loss in this region, including land settlements, logging, illegal cattle ranching, large-scale agriculture (e.g., coffee and palm oil plantations), and subsistence farming [1,22,27,41,42,43,44,45]. In the last two decades, the three largest surviving forest segments in Central America have shrunk in size by about 23% and are limited to a few pockets of old-growth forests mostly bound by international borders and heavily urbanized regions. Within these forests, there are indigenous populations and biodiverse ecosystems; however, as climate change intensifies, urban areas expand, and clearing for farmland continues, these forests will continue to shrink [28,38]. 

From 2001 to 2010, an average of 5376 square kilometers (2076 sq mi) of forest disappeared in the region. Currently, the percentage land area covered by forests in Central America varies by country, with the highest in Costa Rica (58.8%), followed by Honduras (57.2%), Panama (57.1%), Belize (57%), Guatemala (33.1%), Nicaragua (30%), and El Salvador (28.6%) (Table 1) [46]. Most of the deforestation in Central America is in the moist forest biome of the Caribbean slopes of Nicaragua [44]. Recent reports have revealed that over 90% of forest loss in Central America is due to extensive illegal cattle ranching with most of it occurring in Indigenous territories and protected areas. This illegal activity is often connected to money laundering and drug trafficking [22,38,45]. Other agricultural activities, such as the proliferation of oil palm plantations, have displaced cattle and people into protected areas, which further accelerates deforestation in the region. Regions such as La Mosquitia in Nicaragua and Honduras and the Maya Forest region located between Mexico, Guatemala, and Belize are under the greatest threat [22,38].

Deforestation rates in protected areas differ among environmental governance models and intensity of human activities [47,48,49]. Recent studies indicated that drug trafficking and related criminal activities have significantly contributed to forest loss in Central America since the early 2000s [22,45,50]. As a result of successful and disruptive U.S.-led interdiction activities in the Caribbean and Mexico, illegal drug traffickers were forced to diverge cocaine shipments through Central America, which is currently the primary trafficking corridor for cocaine between South to North America [50,51,52]. Presently, about 86% of the cocaine trafficked worldwide is transported across Central America, leaving billions of dollars in annual illegal profit in the region. Moreover, approximately 10% to 14% of the gross domestic product of Nicaragua, Honduras, and Guatemala, a major drug corridor in the region, is linked to illegal drug trafficking [22,45,52]. In these three countries, where most of the Central American forest loss has occurred, drug trafficking along with other illicit trades are increasingly cited as principal drivers of environmental degradation, accounting for about 25% of all forest loss since the mid-2000s [53,54,55]. These regions are characterized by poor socioeconomic development located in remote forests that are highly vulnerable to deforestation [45,50,52,54].

Effective drug smuggling territories are characterized by their remoteness and protected forests status cutting across international terrestrial and marine borders and are seldom monitored by national drug enforcement agencies. Moreover, these smuggling territories typically have weak civil governance structures, insecure land tenures, high unemployment, and are frequently controlled by low-resource conservation groups and agencies that are highly susceptible to undermining and exploitation by criminal organizations [22,45,54,56,57,58]. The increasing use of these protected areas for illicit activities diminishes the region’s capacity for conservation of forest cover and biodiversity that could help reduce the effects of climate change in the region [29,45]. When cocaine is trafficked through Central America, money is laundered through the conversion of forests to agricultural land in order to legitimize illicit profits in the legal economy. Money laundering activities linked to deforestation include illegal cattle ranching and timber extraction, clandestine airstrips, and mining. The most destructive of these illegal activities is cattle ranching [22,38,45,50,53,55,59]. Protected and remote areas highly impacted by drug trafficking and related illicit activities include the Petén and Nicaragua’s Caribbean Coast, Guatemala’s Maya Biosphere Reserve, and Honduras’s Rio Plátano Biosphere Reserve [44,45,55,57,60,61,62,63].

In Central America, drug trafficking has produced distinctive patterns of extensive deforestation and other forms of environmental degradation [45,62,63]. Deforestation in this region is considered a large-scale, late-stage effect of ‘‘narco-degradation” that reflects changes in smuggling routes and greatly varies in the time, space, type, and intensity of these activities, typically emerging at a local level as drug trade inserts in specific locations across Central American countries [22,45].

## 3. Urbanization in Central America

Central America is currently experiencing a major demographic transition along with an accelerated growth of urban populations. National statistics suggest that, between 2000 and 2014, rural population growth in this region has been declining while urban populations have been steadily increasing [45]. After Africa, Central America is the second-fastest urbanizing region in the world [30]. Over the past 20 years, Central American urban populations grew at an average rate of 3.8% per year, which is twice as fast as other Latin American populations and 1.7 times faster than the global average. Although the proportion of urban populations increased to 59% today, compared to 48% in 1990, Central America remains the least urbanized region in Latin America. As a consequence of rural-to-urban migration and natural population growth, it is expected that the urban population of Central America will double by the year 2050, growing to more than 25 million new urban inhabitants [30]. Central American countries with the highest-to-lowest percentage of urban populations are Costa Rica (81%), El Salvador (73%), Panama (68%), Nicaragua (59%), Honduras (58%), Guatemala (52%), and Belize (46%) (Table 1) [30,64].

Across countries, official definition differences of what “urban” constitutes make comparisons between countries difficult. In Central America, the definition of urban varies widely. For example, in Guatemala and Honduras, any human settlements with a population larger than 2000 residents with access to basic infrastructure, such as electricity and piped water, are classified as urban. In Panama and Nicaragua, urban is defined as settlements of 1000 and 1500 inhabitants, respectively, Moreover, in Costa Rica and El Salvador, urban areas are defined as people that are living within municipal boundaries (locally known as “cantones” or “cabeceras municipales”), regardless of population size [30].

In Central America, large numbers of poor, rural populations are migrating to cities in search of better educational and employment opportunities and improving their quality of life. The most dominant factors driving this migration include declining agricultural prices, environmental degradation, vulnerability to natural disasters, food insecurity, violence, and economic instability [30]. This large influx of migrants poses significant challenges for cities, including the provision of adequate urban infrastructure and reliable basic services, such as sewer, water, and waste management, the worsening of existing housing deterioration, and increased vulnerability to natural disasters [30,65].

Although cities contain most of a country’s population, recent population expansions in Central America are driven by growing urban agglomerations in secondary cities in areas surrounding capital cities. For instance, most urban population growth in Costa Rica and Guatemala has taken place outside the capital cities [30]. Moreover, recent trends show that land development in the region has been intensifying faster than population growth. This expansion of built-up areas results in sprawling urbanization, which accelerates deforestation [30,66]. In rural areas, population growth has also been a major driver of environmental change, which further exacerbates deforestation, impacts land use, and changes animal husbandry practices [30].

Unplanned and uncontrolled urbanization, characterized by the development of informal settlements in high-risk areas with deficient building standards and infrastructure and increase flood risks, has led to increased vulnerability to natural disasters in the region [67,68]. Large-scale flooding is the most common disaster with close to 40 events occurring across the Central America between 2006 and 2010 [30]. Furthermore, storms have frequently impacted the region, including Hurricane Mitch in 1998 which directly affected about 6.7 million people, causing 14,600 deaths and over USD 8.5 billion in damages in Honduras, El Salvador, Nicaragua, and Guatemala [30]. Recently, back-to-back category 4 storms, Eta and Iota, devastated much of the region and impacted nearly 7 million people in November of 2019 [30,69,70]. It is expected that climate change will further modify current weather patterns in Central America potentially leading to an increase in the number and severity of extreme meteorological events. Increase frequency and intensity of floods, droughts, and hurricanes could affect access and quality of water and alter ecosystem services in affected areas [68,71].

## 4. Mosquito-Borne and Tick-Borne Diseases in Central America

The global emergence and resurgence of VBDs in the last three decades are closely linked to demographic, economic, and societal changes. The decay in public health infrastructure required to prevent and control these diseases and the unprecedented population growth, primarily in rapidly growing cities, are factors that have facilitated VBD transmission and their geographic spread [2,5]. In Central America, the most important VBDs affecting humans and animals include Chagas disease, leishmaniasis, dengue fever, malaria, Zika fever, chikungunya fever, West Nile fever, rickettsial diseases, Eastern equine encephalitis, Saint Louis encephalitis, and Venezuelan equine encephalitis (Table 2) [6,72].

### 4.1. Mosquito-Borne Arboviral Diseases in Central America

#### 4.1.1. West Nile Virus Disease

West Nile virus (WNV) (genus *Flavivirus*, family *Flaviviridae*), initially discovered in Uganda in 1937, has become endemic in parts of the Americas since its introduction in 1999 [73]. In Central America, the first evidence of its active circulation in horses was detected between November 2001 and April 2003 in El Salvador, where 203 horses died from undetermined encephalitis [74], and Belize, where a single encephalitic horse was diagnosed with WNV infection [75]. In addition, ten serum samples from horses in the same area were positive for WNV. Although no human cases were reported during this outbreak [74], another study carried out in 2003 in Guatemala, Belize, and El Salvador detected antibodies against the virus in humans [76,77]. Moreover, in 2006, a single case of WNV infection was detected in a Spanish missionary who was living in Nicaragua [78].

Several studies have been conducted in Central America to understand WNV transmission involving equines and mosquitoes. For instance, studies were conducted in Guatemala to determine WNV transmission dynamics and seroepidemiology [79,80,81]. Of the seven departments selected for monitoring by sentinel chickens in the country, one transmission focus was identified in the eastern city of Puerto Barrios. Annual transmission at that site was detected between the months of May and October of 2005–2008. Additionally, great-tailed grackles (*Quiscalus mexicanus* [Gmelin, 1788]) have been identified as primary amplifying hosts in the region [79,81]. Environmental factors, such as high temperatures and low rainfall, are strongly associated with chicken seroconversions since these factors influence mosquito population density and viral infection kinetics in the vector in both rural and urban environments [79]. Another study, conducted between 2003–2004, evaluated 352 Guatemalan horses for WNV antibodies finding nine horses positive for WNV, 33 for SLEV, and 21 positive for undifferentiated flaviviruses [80]. Recently in Panama, a study by Carrera et al. (2018) found a WNV seroprevalence in equids with neurological disease of 2.6% [82].

In Costa Rica, WNV circulation was first detected in 2004 in seropositive horses from the Guanacaste Province with a prevalence of 18–28%, followed by the first equine with neurologic disease in 2009 in the same region [83]. Since then, new equine cases are reported annually in the country, especially in the lowlands during the rainy season [84]. Serological evidence of WNV infection in wildlife has also been reported, including non-human primates, Hoffman’s two-toed (*Choloepus hoffmanni* Peters, 1858) and brown-throated sloths (*Bradypus variegatus* Schinz, 1825), and birds [79,80,85,86,87]. The ecological distribution of these species coincides with the geographic distribution of neurological cases in equines [84,86,87]. The importance of wildlife in the enzootic transmission of WNV in Central America is largely unknown. A more recent study by Piche-Ovares et al. (2021) during the rainy and dry seasons reported additional serological evidence of neutralizing antibodies against WNV in equines, humans, sentinel chickens, a local wild bird, and one seroconversion event in a horse [88]. However, the study did not find molecular evidence of active virus circulation in wild birds and mosquitoes. Although human cases of neurological WNV infection have not yet been recorded in Costa Rica, evidence suggests that the virus is widely distributed throughout the region [88].

A limited number of studies have examined the incrimination of mosquito species in WNV transmission in Central America. For instance, a study by Morales-Betoulle et al. (2013) at a WNV transmission foci in Guatemala isolated the virus from *Culex quinquefasciatus* Say, 1823 and *Culex mollis* Dyar and Knab, 1906/*Cx. inflictus* Theobald, 1901 mosquitoes; however, no isolates were obtained from the most abundant species, *Cx. nigripalpus* Theobald, 1901 [79]. Laboratory vector competence using Central American WNV strains and mosquitoes have found evidence of moderate-to-strong competency for *Cx. quinquefasciatus* and *Cx. nigripalpus* in Honduras and Guatemala [89,90].

One of the most critical issues in Central America is the lack of data on the extent of WNV human disease burden in the region since it can be very challenging to diagnose these infections. Human WNV cases are often misdiagnosed or underdiagnosed due to several factors, including serological cross-reactivity with other flaviviruses circulating in the region, virus genome diversity, low transient viremia, and the lack of laboratory testing capacity [91]. Presently, the diagnosis of WNV infection is mostly based on serological methods since molecular identification of virus RNA is often unreliable due to the short-term transient viremia and low viral load at the time of the onset of symptoms [92]. As seen in other areas where the WNV circulates, the serological diagnosis of WNV disease in Central America is also problematic due to the active co-circulation of dengue virus, Zika virus, and other flaviviruses that will likely produce cross-reactivity in serological assays [92]. Lastly, laboratory capacity to test for WNV and other VBDs varies widely among Central American countries, ranging from some laboratory capacity and epidemiological surveillance systems to a lack of critical resources, such as appropriate testing technologies, reagents, facilities, epidemiological surveillance systems, and technical expertise [93].

One of the possible reasons for the lack of evidence for human and equine WNV neurological disease and high avian mortality in Central America is that birds infected with more virulent strains could not start their migration process to South America while only birds infected with less virulent viral strains are able to migrate [94]. Another hypothesis that seeks to explain the relatively low human, equines, and avian mortality in Central America focuses on pre-existing neutralizing antibodies against other flaviviruses, such as dengue virus (DENV) and Saint Louis encephalitis virus (SLEV), which might offer partial protection from WNV disease [77,95,96]. Finally, environmental and host factors, such as temperature, vector competence, and host susceptibility, may influence the genetic selection of less virulent variants [95]. Although WNV disease has not been reported in most of Central America, all the components that could support virus circulation are present throughout the region maintaining the risk for future outbreaks in the region.

#### 4.1.2. Saint Louis Encephalitis

Saint Louis encephalitis virus (SLEV) (genus *Flavivirus*, family *Flaviviridae*) was first identified in St. Louis, Missouri, USA in 1933. SLEV can be classified into eight genotypes, with genotypes I and II circulating mainly in the US and associated with outbreaks of human encephalitis [97], while genotypes III–VIII have been found only in Central and South America. In Central America, SLEV circulation was first documented in 1957 in Buena Vista, Panama, when the virus was isolated from a pool of *Sabethes chloropterus* (von Humbolt, 1819) [98]. Subsequently, serological evidence and isolates of SLEV were also detected in Maje Island, Darien Province, and Panama Province in humans, wild birds (local and migratory), several wild mammals (rodents, non-human primates, sloths), various sentinel animals, and several mosquito species, including *Haemagogus lucifer* (Howard, Dyar and Knab, 1913), *Deinocerites pseudes* Dyar and Knab, 1909, *Mansonia dyari* Belkin, Heinemann and Page, 1970, *Cx. nigripalpus*, *Trichoprosopon* spp., and *Wyeomyia* spp. [98,99,100,101]. In the Pacific coast of Guatemala, the virus was also isolated from *Cx. nigripalpus* [102].

More recently, two studies in Costa Rica reported serological evidence of SLEV infection in free range and captive non-human primates and sloths. For example, a study by Chavez et al. (2021) found homotypic and heterotypic neutralization reactivity to SLEV of 47.6% and 5.9%, respectively, in mantled howler monkeys (*Alouatta palliata* [Gray, 1849]) and spider monkeys (*Ateles geoffroyi* Kuhl, 1820), while another study found a seropositivity of 42% in Hoffman’s two-toed sloths (*C. hoffmanni*) and brown-throated sloths (*B. variegatus*) [85,86]. Moreover, a study conducted between 2017 and 2018 in northwestern and southeastern Costa Rica found neutralizing SLEV antibodies in humans, local and migratory birds, and equines [88]. One seroconversion event to SLE, between the rainy and the dry seasons, was also detected in a horse from the province of Guanacaste [88].

#### 4.1.3. Venezuelan Equine Encephalitis

Venezuelan equine encephalitis virus (VEEV) (genus *Alphavirus*, family *Togaviridae*) is a serocomplex of six antigenic subtypes (I–VI). Within subtype I, there are five antigenic variants (AB–F), with antigenic variants IAB and IC associated with epizootic/epidemic activity in equines and humans, while variants ID, IE, and IF and subtypes II–IV are associated with enzootic forest cycles. These enzootic viruses circulate in sylvatic rodent populations within tropical and subtropical forests or swamp habitats continuously transmitted by *Cx. (Melanoconion)* mosquitoes [103,104]. Most enzootic variants are avirulent in equines, but some strains could cause clinical disease in both humans and equines with a similar clinical presentation to epizootic strains infections [105].

The first VEEV epizootic involving humans and equines in Central America took place between 1969–1970 in Honduras, Guatemala, Nicaragua, El Salvador, and Costa Rica, as part of a major intercontinental epizootic that spread from Central America to northern South America and south Texas, U.S. [106,107]. The virus strain responsible for this epizootic was identified VEEV subtype IB (later reclassified as IAB) and, at the time, was commonly isolated from equines, humans, and several mosquito species, including *Psorophora confinnis* (Lynch Arribalzaga, 1891), *Cx. nigripalpus*, *Cx. (Melanoconion)* sp., *Ma. titillans* (Walker, 1848), *Aedes taeniorhynchus* (Wiedemann, 1821), and *De. pseudes* [106,108,109,110]. Following this epizootic, numerous studies linked the origin of the VEEV epizootic subtype IAB to incompletely innactivated vaccines [111]. Moreover, evidence also suggest that key mutations in enzootic VEEV strains could potentially mediate the emergence of novel epizootic strains [112].

In recent decades, only two enzootic VEEV subtypes have been reported in Central America. The VEEV subtype IE appears to be widely distributed throughout the region while the VEEV subtype ID has been detected only in Panama [82,84,112,113,114]. Recent data suggest that humans infected with these two enzootic subtypes could develop sufficient viremia to potentially infect both Central American enzootic and epizootic VEEV vectors [112,113]. Moreover, experimental competency studies suggest the potential transmission of VEEV subtype ID in Central America by *Ae. aegypti* (Linnaeus, 1762) and *Ae. albopictus* (Skuse, 1894), increasing the possibility that urban transmission cycles could take place in the region [115,116]. It is possible that febrile illness linked to endemic VEEV infections in Latin America are more widespread than previously observed but are vastly unrecognized or misdiagnosed due to the presence of similar dengue-like febrile illnesses and lack of effective public health surveillance systems [112,113].

The first reported human case of enzootic VEEV ID virus infection in Panama took place in the early 1960s near Panama City [117]. Recently, an increase in human VEEV cases in Panama have intensified ecological and epidemiological studies in the region, with several studies reporting the active and stable circulation in humans of both VEEV subtypes ID and IE throughout the country. For example, recent serosurveys have found neutralizing VEEV antibodies levels between 8.5% and 78% across several communities in the Darien region [82,113,114]. Moreover, subtype ID outbreaks in Panama also show higher case fatality rates than those reported during previous subtype IAB epidemics [113].

Field studies in Panama, Costa Rica, Guatemala, Belize, and Honduras have also detected the circulation of enzootic VEEV ID and IE viruses in a variety of vertebrates, including wild rodents (*Zygodontomys brevicauda* [Allen and Chapman, 1893]), *Transadinomys (=Oryzomys) bolivaris* Allen, 1901, *Proechimys semispinosus* [Tomes, 1860], *Melanomys caliginosus* [Tomes, 1860]; *Oryzomys* spp.), opossums (*Didelphis marsupialis* Linnaeus, 1758, and *Marmosa robinsoni* Bangs, 1898; *Philander* spp.), sloths (*Bradypus* spp. and *Choloepus* spp.), sentinel guinea pigs and hamsters, equines, wild and domestic birds, and bats [82,84,114,118,119,120,121,122,123,124]. Moreover, entomological surveys in Central America have identified *Cx. (Melanoconion)* spp., particularly *Cx. (Mel.) taeniopus* Dyar and Knab, 1907, *Cx. (Mel.) ocossa* Dyar and Knab, 1919, and *Cx. (Mel.) panocossa* Dyar, 1923; formerly known as *Cx. (Mel.) aikenii* (Aiken and Rowland, 1906), *Cx. (Mel.) vomerifer* Komp, 1932, and *Cx. (Mel.) erraticus* (Dyar and Knab, 1906) as the most important enzootic VEEV vectors in the region [112,125].

#### 4.1.4. Madariaga Virus

Maradiaga virus (MADV) is an emergent *Alphavirus* (family *Togaviridae*) in the Eastern equine encephalitis (EEE) antigenic linage III strain complex. This virus was previously known as the EEE South American variant and it is maintained in stable, enzootic cycles throughout Central and South America [126]. Although broad spectrum human and equine infections linked to MADV have been reported [124,127,128], a recent outbreak in Panama underscores concerns as an emergent virus in Latin America [114,129]. Although the enzootic cycle of MADV remains unclear, its circulation has been detected in birds, rodents, marsupials, reptiles, and bats [130]. In Panama, several rodent and bat reservoirs have been proposed, including the black rat (*Rattus rattus* [Linnaeus, 1758]), short-tailed cane mouse (*Z. brevicauda*), long-whiskered rice rat (*T. bolivaris*), Tome’s spiny rat *(P. semispinosus*), Seba’s short-tailed bat (*Carollia perspicillata* [Linnaeus, 1758]), and pale spear-nose bat (*Phyllostomus discolor* Wagner, 1843) [114]. The primary mosquito vectors for this virus are *Cx. (Melanoconion)* mosquitoes, especially *Cx. (Mel.) taeniopus*, which could serve as an enzootic and epizootic vector [130]. In Central America, MADV isolates have been obtained also from *Cx. (Mel.) taeniopus* in Panama during field surveys and equine outbreaks [127,131].

The first report of an outbreak of MADV-related neurologic disease took place in 2010 in Darien, Panama, where seven humans and 210 horses developed encephalitis and were confirmed positive for the virus [129]. In 2017, another outbreak was reported in the same region of Darien. A serosurvey conducted in the area showed a higher seroprevalence than during previous investigations [132,133]. Human activities, such as horse and cattle ranching, fishing, farming, pasture, and poor housing conditions, have been identified as risk factors associated with MADV infections [114]. Moreover, people living near or having vegetation around the house had higher seroprevalence to MADV [133]. Therefore, the increased exposure of people to MADV in this region could have resulted from ecological changes, primarily deforestation, which increased human contact with enzootic transmission cycles. The co-circulation of MADV and VEEV makes diagnosis difficult in regions where these viruses are endemic [129].

#### 4.1.5. Yellow Fever

Yellow fever virus (YFV) is a member of the genus *Flavivirus* (family *Flaviviridae*) primarily transmitted by the bite of *Aedes (Stegomyia*) spp., *Haemagogus* spp., and *Sabethes* spp. mosquitoes in tropical and subtropical regions of Africa and South America [134]. In the Americas, YFV is currently distributed between southern Panama and northern Argentina [134,135]. In Central America, outbreaks have been recognized as far back as the mid-1600s in the Yucatan Peninsula and through the construction of the Panama Canal in the late 19th century [136,137,138]. From 1905 to 1948, there was a period of relative quiet with no autochthonous cases of yellow fever reported; however, between 1949 and 1954, a large human outbreak of sylvatic YF, which originated in Panama, spread northward to Costa Rica, Nicaragua, Honduras, Guatemala, and the Guatemala–Mexico border [139]. During this period, a high mortality in non-human primates was also reported in the region [138,140]. At the time, several entomological surveys conducted in Costa Rica and Nicaragua identified a high abundance of *Haemagogus* spp. and *Sabethes* spp. mosquitoes in the region affected by the epizootic [141,142,143]. In addition, the virus was isolated in Panama from *Hg. lucifer*, *Hg. equinus* Theobald, 1903, *Hg. spegazzinii* Brethes, 1912, and *Sa. chloropterus*, and in Guatemala from *Hg. mesodentatus* Komp and Kumm, 1938, *Hg. equinus*, and *Sa. chloropterus* during the same time period [144,145]. The vector competence of Guatemalan and Panamanian *Haemagogus* and *Sabethes* species for YFV was established via a mouse inoculation experiment [146]. The presumptive reservoir of YFV in Central America is the howler monkey (*Alouatta* spp.), which is highly susceptible to the infection and has shown high mortality levels during epizootics [138]. After the 1950s, no other urban outbreaks of YF were documented in Central America; however, sporadic outbreaks of sylvatic yellow fever took place during the 1960s in the region [147].

#### 4.1.6. Zika Fever

Zika disease (or Zika fever), caused by the Zika virus (ZIKV, family *Flaviviridae*, genus *Flavivirus*), first detected in Uganda in 1947 [148], has received intense scrutiny since its emergence as a significant human pathogen in recent years [149,150,151]. ZIKV is now considered endemic throughout Latin America [152]. In Central America, the first autochthonous cases of Zika fever were documented in November 2015 in El Salvador and Guatemala [153]. The virus rapidly spread through the rest of Central America, with cases reported in Honduras and Panama and later in Costa Rica and Nicaragua [154,155]. By April 2016, ZIKV was present in all Central American countries, with Belize being the last in which introduction was documented [156]. Early into the epidemic, two ZIKV strains obtained in Guatemala in 2015 were sequenced and identified as belonging to the Asian lineage, the same lineage detected in Brazil that was spreading to other countries that year [157]. However, phylogenetic analyses have suggested that ZIKV was likely introduced from Brazil to Honduras as early as late 2014 and spread undetected to other Central American countries [158]. Overall, the rapid introduction and spread of ZIKV through Central America probably resulted from the constant movement of people between these countries as well as environmental and climatic conditions that promote high densities of *Ae. aegypti* and can drive transmission dynamics [159,160]. Most cases of Zika fever in the region have been reported in El Salvador, Belize, Nicaragua, and Honduras [161].

Although Zika fever incidence has decreased in the past few years, it is still one of the most frequent arboviral diseases in Central America. The overlapping signs and symptoms of dengue fever, Zika fever, and chikungunya fever, as well as the unavailability of widespread laboratory confirmation in many areas of Central America, make diagnosis and epidemiological surveillance of these diseases challenging [162,163]. Moreover, several studies suggest that ZIKV infection rates during the recent American epidemic provided adequate herd immunity to lessen the risk of another large epidemic for at least another 10 years [164]. Nonetheless, the scale of ZIKV transmission has remained patchy and widely variable in the Americas [165].

The establishment of sylvatic cycles involving non-human primates has not been reported yet in the Americas; however, its possibility cannot be ruled out [166]. There is consensus that ZIKV can potentially become established in sylvatic cycles between non-human primates and mosquitoes; however, field evidence is still inconclusive [167]. At least three primate species present throughout Central and South America [168], Ma’s night monkey (*Aotus nancymaae* Hershkovitz, 1983), Guianan squirrel monkey (*Saimiri sciureus* [Linnaeus, 1758]), black-tufted marmoset (*Callithrix penicillata* [Geoffroy, 1812]) are susceptible to ZIKV infection. Although they usually do not develop clinical symptoms, their viremia can potentially support transmission based on experimental infections [169,170]. Regarding potential sylvatic vectors for ZIKV in Central America, experimental studies have shown that *Sa. cyaneus* (Fabricius, 1905) is a competent vector, but less competent than *Ae. aegypti*, while *Ha. leucocelaenus* (Dyar and Shannon, 1924) have shown low rates of dissemination. [171,172].

#### 4.1.7. Chikungunya

Chikungunya virus (CHIKV) (family Togaviridae, genus Alphavirus), the etiological agent of chikungunya fever, was first identified in Tanzania in 1952, and has recently spread throughout tropical and subtropical regions of the world, including the Americas [173,174]. In 2014, the first cases of CHIKV in Central America were reported in El Salvador followed by Guatemala, Costa Rica, Honduras, and Nicaragua [175,176,177]. In Panama, recent studies reported CHIKV seroprevalence strongly associated with densely populated urban and periurban areas, poor socioeconomic conditions, and high infestation indices of the vectors *Ae. aegypti* and *Ae. albopictus* [178,179]. Similar results have been found in Nicaragua, where a serosurvey involving over 11,000 participants, found 39% CHIKV seroprevalence associated with sites containing high vector infestation indices [180].

The potential introduction of sylvatic cycles of CHIKV in the Americas is still under investigation. Although serological evidence is weak, there is potential for the introduction of CHIKV sylvatic cycles through spillback events [181]. For instance, experimental studies have shown that some reptiles and amphibians maybe susceptible to infection [182], while there is limited evidence on the role of neotropical non-human primates in the establishment of CHIKV sylvatic cycles [183]. Regarding potential vectors, *Ha. leucocelaenus* and *Ae. terrens* (Walker, 1856), two sylvatic species of mosquitoes in the neotropics, appear to be competent experimental vectors of CHIKV [184]. It has been suggested that the level of herd immunity recently observed throughout much of the Americas could limit the occurrence of major new epidemics until the next population generation provide additional amplifying hosts [165].

#### 4.1.8. Dengue Fever

Dengue fever, a disease caused by four dengue viruses (DENV, genus *Flavivirus*, family *Flaviviridae*) serotypes, emerged and evolved from sylvatic cycles in Asia and are primarily transmitted to humans by *Ae. aegypti* mosquitoes. *Aedes albopictus* could also act as a DENV vector, particularly in areas where *Ae. aegypti* is absent; however, it has been difficult to directly incriminate *Ae. albopictus* as a DENV vector during autochthonous arbovirus outbreaks [179,185,186,187]. Moreover, recent evidence suggests that this species maybe effective as a natural reservoir of DENV via transovarial transmission [188].

The first reports of dengue fever in Central America were made in the early 20th century in Panama [189], although the occurrence of human cases could go as far as the 1600s [190]. The failure to eradicate *Ae. aegypti* populations in the 1960s and 1970s triggered a resurgence of dengue fever in the Americas [190]. Between 1978 and 1980, an increase in dengue fever cases was observed in Guatemala, Belize, and El Salvador [191,192]; then, in 1985, a major epidemic in Nicaragua caused by DENV 1 and DENV 2 affected over 17,000 people [193]. In 1993, Costa Rica and Panama confirmed local transmission and autochthonous cases of dengue fever for the first time in 40 to 50 years [194]. Since then, the virus has become endemic in both countries [195,196]. Further details on the first reports of different DENV subtypes in Central America can be found elsewhere [190,191,197]. The re-emergence of dengue fever in Central America, just as in other parts of the Americas, has been attributed to several factors, including diminished political importance in countries where eradication was achieved, reduction in surveillance and other public health resources, development of insecticide resistance and resurgence of *Ae. aegypti* populations, establishment of *Ae. albopictus*, social disparities, and increased urbanization in the region [179,190,198,199,200].

Today, there is little evidence that establishes the existence of sylvatic DENV transmission cycles in the Americas. Although several neotropical non-human primate species, including white-faced capuchin monkeys (*Cebus capucinus* [Linnaeus, 1758]), spider monkeys (*Ateles* spp.), and mantled howler monkeys (*A. palliata*), are susceptible to DENV infection, serological surveys of Panamanian monkeys failed to show evidence of enzootic circulation [201,202]. Recently, serological and molecular evidence of DENV infection was reported in several species of non-human primates in Costa Rica, which suggest potential bidirectional exposures due to the presence of bridging vectors or an increase in human–wildlife contacts [85,87].

#### 4.1.9. Mayaro Fever

Mayaro virus (MAYV) is a neotropical *Alphavirus* (family *Togaviridae*) member of the Semliki Forest antigenic complex initially isolated in Trinidad in 1954 [203]. It has been detected throughout the Americas, from Mexico to Brazil and the Caribbean [204], and it is considered a neglected viral disease in humans [203,204]. Infection with MAYV is characterized by a self-limiting febrile illness accompanied by long term incapacitating arthralgia [203]; however, severe cases and even deaths have also been reported [204]. The virus circulates in continuous sylvatic cycles between canopy dwelling *Hg. janthynomis* Dyar, 1921 and non-human primates, including howler monkeys (*A. seniculus* [Linnaeus, 1766], *A. caraya* (Humbolt, 1812), and *A. villosa* [Gray, 1845]), silvery marmosets (*C. argentata* [Linnaeus, 1771]), and capuchin monkeys (*Sapajus* spp.). Human cases are typically associated and restricted to the edges of neotropical rain forests causing limited outbreaks [204,205]. Sylvatic cycles may also include other animals, such as sloths, sheep, rodents, horses, reptiles, agoutis, and birds, but their role in transmission is still unclear [206,207]. Vector competence for MAYV and numerous field isolates have also been reported with other mosquito species, including *Anopheles* spp., *Culex* spp., *Sabethes* spp., *Mansonia* spp., and *Psorophora* spp. [206,208]. In Central America, very little is known about the epidemiology and ecology of MAYV and its potential for emergence as an important pathogen. Only a small number of field studies have been conducted in Central America. In Panama, Guatemala, and Costa Rica, MAYV has been detected in humans, sentinel monkeys, wild howler monkeys (*A. villosa*), and agoutis (*Dasyprocta punctata* Gray, 1842) [82,132,205,206,209,210,211], while in mosquitoes, the virus has been isolated in Panama from *Ps. ferox* (Humbolt, 1819) and *Cx. (Mel.) vomerifer* [118,212].

Disease spillover into rural and peri-urban areas has been reported and there is increasing concern that MAYV could become urbanized since it could adapt to replicate in the urban vectors *Ae. aegypti* and *Ae. albopictus*, whose competence has been reported in both the laboratory and the field [204,213,214]. The number of true MAYV cases in the Americas is potentially higher than what has been reported due in part to potential misdiagnosis and underdiagnosis, clinical similarities to other arboviral infections, and coinfections with other endemic arboviruses, such as DENV and CHIKV [204].

### 4.2. Malaria in Central America

Malaria, a febrile disease caused by *Plasmodium* spp. parasites transmitted by *Anopheles* spp. mosquitoes, causes the highest morbidity and mortality compared to any other VBD worldwide [215]. While less than 1% of the 2019 global malaria burden was recorded in the Americas, the region remains endemic for the disease. In Central America, there were nearly 20,000 autochthonous malaria cases recorded in 2019, with cases reported in five out of seven countries within the region: Nicaragua (13,200)), Guatemala (2100), Panama (1600), Honduras (330), and Costa Rica (91) [215]. Almost all these cases were caused by *P. vivax* (Grassi and Feletti, 1890) (74%) and *P. falciparum* (Welch, 1897) (23%), which are transmitted mainly by *An. albimanus* Wiedemann, 1820, *An. pseudopuntipennis* Theobald, 1901, *An. darlingi* Root, 1896, *An. marajoara*-Galvao and Damasceno, 1942, *An. aquasalis* Curry, 1932, *An. albitarsis* Lynch Arribalzaga, 1878, and *An. vestitipennis* Dyar and Knab, 1906, reflecting a remarkable diversity of competent vectors with diverse ecologies and bionomics [215,216,217,218,219,220,221].

For broader context, these 2019 regional case estimates represent more than a 50% reduction from 2010 estimates, when nearly 40,000 cases were recorded, and over 80% reduction from 2000 estimates when more than 340,000 cases were reported [222]. Despite some challenges that persist, current case numbers highlight the remarkable progress and numerous successes achieved by malaria control and elimination programs in the region [223]. For example, El Salvador was recently certified as malaria free while Belize, Costa Rica, Guatemala, Honduras, and Panama have the potential to eliminate malaria within the next five years [224].

While it is important to reflect on this encouraging progress and celebrate recent malaria control successes across Central America, it is equally important for the region not to let its collective guard down. With multiple competent malaria vectors naturally occurring throughout its geography [216], Central America remains vulnerable to the re-introduction of malaria across its entire range. Furthermore, the influx of people from malaria endemic regions of the Americas, both travelers and migrants, as well as international travelers from other malaria endemic regions, creates a low but constant risk of *Plasmodium* parasite re-introduction into areas with greatly reduced local transmission [225,226,227,228]. This risk is exacerbated by the prospects of a changing climate, deforestation, changing land-use patterns, and increasing drug and insecticide resistance [229,230].

### 4.3. Tick-Borne Diseases in Central America

In Central America, recent studies on the ecology of tick-borne diseases are limited for most countries, and outbreaks and case reports in humans are mostly sporadic and infrequent. However, there is evidence of possible zoonotic human pathogens in ticks and/or vertebrate animals, such as *Anaplasma phagocytophilum* (Foggie, 1949), *Ehrlichia canis* (Donatien and Lestoquard, 1935), *E. chaffeensis* Anderson et al., 1992, *E. ewingii* Anderson et al., 1992, *Rickettsia rickettsii* (Wolbach, 1919), *R. parkeri* Lackman et al., 1965; including the strain Atlantic Rainforest), *R. akari* Huebner et al., 1946, *R. africae* Kelly et al., 1996, *Borrelia burgdorferi* s.l. Johnson et al., 1984, and *Borrelia* sp. (causing tick-borne relapsing fever), as well as several other microorganisms that may infect ticks and/or vertebrate animals for which pathogenicity is unknown, or that are not known to cause human disease [231,232,233,234,235,236,237,238,239,240,241,242,243,244,245,246,247,248,249,250,251,252,253,254,255,256,257,258,259,260,261,262,263,264]. In humans, the most common tick-borne diseases reported in Central America in the past few decades are spotted fever group rickettsioses and ehrlichioses, followed by isolated reports of probable Lyme disease in which the bacteria were not directly detected or identified [250,252,265,266,267,268,269,270,271]. In addition, there are records of tick-borne relapsing fever and serological evidence of exposure to *R. akari* [235,248,272], while human anaplasmosis and *R. parkeri* spotted fevers have yet to be confirmed in the region. In most cases of outbreak descriptions of human tick-borne diseases, the main vectors and vertebrate reservoirs implicated in local transmission cycles have not been clearly identified.

#### 4.3.1. Rickettsioses

Bacteria of the genus *Rickettsia* (Rickettsiales: family *Rickettsiaceae*) are responsible for various clinical rickettsioses in humans; in the Americas, *R. rickettsii* spotted fever is the most relevant in terms of morbidity and mortality [273]. Tick-borne rickettsioses have been known to occur in Central America since the 1950s [252,274]. The first cases were identified and confirmed by isolation of *R. rickettsii* from humans and ticks in Panama and later in Costa Rica [252,271]. Ecological investigations to identify possible vertebrate hosts and tick vectors have been carried out in these countries throughout the decades. For instance, *Amblyomma mixtum* Koch, 1844 (previously referred to as *A. cajennense*) has been implicated as a probable vector of *R. rickettsii* to humans in Panama and probably Costa Rica [252,271]. This agrees with the information available about transmission of rickettsiae in South America, where several tick species belonging to the “*A. cajennense* species group” are considered vectors of *R. rickettsii* [275,276]. Moreover, the brown dog tick, *Rhipicephalus sanguineus* s.l. (Latreille, 1806), has also been investigated as a possible vector of urban human cases of *R. rickettsii* infection in Panama [252,277]. As for other spotted fever group rickettsiae, there are reports of human disease and outbreaks in Guatemala, Honduras, Nicaragua, although the species was not identified in these cases [278,279,280]. In addition, there are historical records in which antibodies against *R. akari* (or an antigenically similar species) have been detected in humans in Central America, but there are no records of direct detection of the bacterium in ticks or human cases of rickettsial pox [272]. *Rickettsia akari* transmission is usually associated with hematophagous mites, but it has been detected in humans, dogs, and ticks in neighboring Yucatan, Mexico [281,282]. Recently, DNA of *R. africae* was detected in *A. ovale* Koch, 1844 ticks from Nicaragua, but there are no human cases of African tick-bite fever confirmed to this date [254].

#### 4.3.2. Ehrlichiosis and Anaplasmosis

The most relevant species of *Ehrlichia* and *Anaplasma* (Rickettsiales: family *Anaplasmataceae*) in terms of morbidity in humans are *E. chaffeensis* and *A. phagocytophilum*, which cause human monocytic ehrlichiosis and human granulocytic anaplasmosis, respectively [283,284]. Different species of *Ehrlichia* and *Anaplasma* are known to occur in Central America, especially in domestic animals (e.g., *E. canis*, *E. chaffeensis*, *Ehrlichia* sp. H7, *A. platys* (Dumler et al., 2001), *A. phagocytophilum*), while *E. ewingii* have been detected only through DNA in ticks [231,234,236,237,238,239,241,242,243,244,246,253,255,257,285]. In humans, there are several reports of possible ehrlichiosis/anaplasmosis diagnosed by observation of morulae in stained blood smears, by indirect antigen or antibody detection, without isolation, or by molecular identification of the species [250,266,267,269]. In 2015, *E. chaffeensis* was confirmed in humans by polymerase chain reaction (PCR) and DNA sequencing in Costa Rica [245]. In addition, *E. canis* DNA has also been reported in humans in Costa Rica and in one case in Panama, which was the only one associated with severe disease [251,258]. *Anaplasma phagocytophilum* DNA has been detected repeatedly in ticks and domestic animals, but this species has not been confirmed in human infections [234,236,239,249,255,257]. In areas of North America where human ehrlichiosis and anaplasmosis are common, the principal vectors are *Amblyomma americanum* (Linnaeus, 1758) and *Ixodes* spp. (*I. scapularis* Say, 1821; *I. pacificus* Cooley and Kohls, 1943; and others), respectively, but these ticks are not found in Central America or South America, where the possible vectors are still being determined [286,287,288]. As for *E. canis*, studies have confirmed that *R. sanguineus* s.l. is the vector within the dog population in Central America, in correspondence to what is known for other regions [249,286,289].

#### 4.3.3. Borrelioses

The genus *Borrelia* (Spirochaetales: family *Borreliaceae*) includes spirochaetal bacteria that are mostly associated with ticks and reptiles, but also lice [290,291]. Currently, half of the named species (21 of 42) belong to the “relapsing-fever associated” group, and almost all of the other half (20 species) to the “Lyme borreliosis associated” group (*Borrelia burgdorferi* s.l.) [291].

In Central America, the first descriptions of *Borrelia* spp. spirochetes in human blood samples were reported in Panama in 1909 [292]. Although a species of *Borrelia* was not assigned, subsequent studies confirmed that tick-borne relapsing fever was common in humans, and transmission cycles in Panama were associated with argasid ticks that can sometimes feed on humans, such as *Ornithodoros talaje* (Guérin-Méneville, 1849) and *O. rudis* Karsch, 1880 (=O. *venezuelensis*), and on animals such as armadillos and opossums [248,292,293,294]. Later, the bacterium implicated in Panama was presumed to be the same species transmitted by *O. rudis* infecting humans in South America, referred to as *B. venezuelensis* (Brumpt, 1921), although this has not been confirmed with certainty [295]. Unfortunately, no other cases have been reported in the last decades in Panama and the precise identification of the pathogen has not been determined. However, a case of relapsing fever was diagnosed more recently in a traveler who visited areas of Guatemala and the border with Belize, confirming that infections with *Borrelia* spp., and causing relapsing fever, are probably present throughout the region and may be unreported [235,248].

In contrast, *B. burgdorferi* s.l. has been suspected in a few imported and autochthonous human cases in Honduras and Costa Rica, although there is only indirect serological evidence of infection [265,268,270]. The principal vectors of Lyme disease in North America, Europe, and Asia are ticks of the *I. ricinus* species complex, including *I. ricinus* (Linnaeus, 1758), *I. scapularis*, *I. pacificus*, and *I. persulcatus* Schulze, 1930 [296]. These species are not present in neotropical areas and most *Ixodes* spp. in Central America do not frequently bite humans, which supports the idea that Lyme disease does not occur or it is rare in the region [287,297]. Recently, bacterial DNA identified as *B. burgdorferi* s.l. was detected in *Ixodes* c.f. *boliviensis* Neumann, 1904 from Panama, although the ability of this bacterium to infect humans and cause Lyme disease is currently unknown [263].

## 5. Impact of Deforestation, Urbanization, and Changing Land Use Patterns on the Ecology of Mosquito and Tick-Borne Diseases in Central America

### 5.1. Impact on Mosquito-Borne Arboviral Diseases

Major knowledge gaps persist on the enzootic transmission cycles of most arboviral diseases endemic to Central America, including information on geographic distribution, vertebrate reservoir species, mosquito vectors, and human disease risk. Furthermore, little is known about the potential effects of increasing deforestation, forest fragmentation, and urbanization on the ecology and epidemiology of these diseases in the region. While dengue fever has received the most attention throughout Central America [125,298], research on other arboviral diseases in the region have been conducted mostly in Panama and Costa Rica, the countries with highest economic development and significant public health investments in the region [30]. Arboviral diseases present in Central America are of increasing public health concern due to their recent emergence or resurgence and most are considered neglected [6,112,129,203].

Changes in land use can significantly affect mosquito population dynamics, oviposition, abundance, and host-seeking behaviors. Numerous studies have shown that modifications in land use could result in the loss of hosts, predators, and mosquito habitats, which may affect vector population dynamics, abundance, oviposition, and host-seeking behaviors. These environmental modifications could drive mosquito vectors to search for new blood-feeding sources and alternative breeding habitats [3,23,299], promote higher host contact rates, and initiate disease spillover events, introducing new infections into susceptible human populations [300,301,302]. Other factors, such as human migration and urbanization, can significantly impact the distribution and occurrence of arboviruses by driving their emergence or resurgence. Moreover, different sociodemographic factors associated with urbanization, such as social inequality, health care capacity, food safety, population density, and inadequate infrastructure, could be associated with human disease outbreaks [301,303].

Recent studies in the Amazon region have focused on the relationship between deforestation, vector mosquito abundance, and arbovirus outbreaks [299,302,304]. Forest fragments and growing agricultural areas show higher abundance, richness, and diversity of mosquito species. Conversely, mosquito abundance and richness decreased in the urban environment [304,305]. Consequently, anthropophilic species, such as *Ae. aegypti*, could become very efficient vectors in urban areas compared to *Culex* spp. and *Ae. albopictus*, which feed on a broader range of hosts [303]. Moreover, several species that serve as vectors of multiple human pathogens appear to benefit from deforestation, including species found in Central America, such as *An. darlingi, Ae. aegypti*, and *Cx. quinquefasciatus* [300].

Recent studies in central Panama found that mosquito diversity peaks in pristine forest habitats, such as old-growth forests, while the abundance of colonist mosquito vectors (e.g., disturbed-areas specialists) increased significantly in highly disturbed forest sites [306,307]. These differences in mosquito abundances and diversity across various levels of forest disturbance could be attributed to changes in ecological conditions that maybe affecting the quality and availability of larval breeding sites. All together, these results suggest that forest disturbance could drive VBD emergence and increase risk for disease transmission in recently disturbed tropical regions due to the abundance of colonist-vector species. These mosquito vectors tend to be opportunistic feeders, targeting hosts that are readily available [308]. Moreover, a study by Bayles et al. (2020) in Costa Rica found that areas with a high proportion of anthropogenic-altered landscapes, especially in areas with a high degree of agricultural intensification, have the highest transmission risk for VBDs, such as ZIKV, compared to protected areas [309].

The relationship between yellow fever and deforestation was initially established in the first half of the 20th century when ecological observations were made regarding the ability of *Haemagogus* mosquitoes to survive in forests with some degree of deforestation pressure [310]. In recent years, the number of cases of sylvatic yellow fever cases in Brazil have increased significantly in epizootic or transition areas and have been linked to large-scale deforestation and forest fragmentation within urbanized settings [311,312]. The main vectors of YFV in the Americas, *Haemagogus* spp. and *Sabethes* spp., share similar ecological and bionomic characteristics, including acrodendrophilous behavior and opportunistic feeding habits [313]. Studies on these vectors have shown that they are more abundant in sites with lower forest cover suggesting that forest fragmentation could be a critical factor in determining their presence [314]. For example, *Sa. chloropterus* was recently found in deforested areas adjacent to a primary forest in Costa Rica [315], indicating that deforestation could increase microhabitats for mosquito colonization, such as phytotelma and tree holes, typically used by some enzootic yellow fever vectors [142,316,317]. Other studies suggest that deforestation could be pushing YFV vectors to adapt their feeding habits due to pressure from habitat disturbance. For instance, *Hg. janthinomys* Dyar, 1921 and other YFV vectors in Brazil were frequently observed descending to ground level in the presence of humans conducting wood extraction activities or when the non-human primate population number is small [318]. Moreover, others have found that their feeding behavior is more prolonged and aggressive at ground level than in the forest canopy [319]. *Haemagogus* spp. and *Sabethes* spp. are also eclectic feeders able to shift their host seeking among various wild or domestic animals and human hosts according to their local availability, frequently moving vertically from the top of trees to feed at ground level. However, it is not clear if this eclectic feeding behavior is innate or if it has been driven by an increase in deforestation in regions where this species occurs [134,313,320,321]. Although there is no current evidence of active YFV transmission by *Ae. aegypti* or *Ae. albopictus* [322], experimental studies have determined that local domestic and peridomestic mosquito populations may be competent vectors for YFV strains circulating in South America [323].

The recent reemergence and dispersion of yellow fever in Brazil, potentially driven forest fragmentation near urbanized areas [312], raises the possibility of its resurgence elsewhere in the continent, including parts of Central America, where these sylvatic vectors are also present and similar ecological disturbances are taking place. The urbanization of yellow fever is one of the biggest infectious disease threats in Latin America [324]. The establishment of urban cycles, via *Ae. aegypti and Ae. albopictus*, could be catastrophic since these vectors are widely distributed throughout the region [318]. Most people living in urban areas of Latin America are not vaccinated, which could lead to high disease incidence that could further spread transmission [325]. It has been estimated that the proportion of infected people during an epizootic could be up to 29% under these conditions [326].

Deforestation can also affect non-human primate reservoirs of YFV and other arboviruses in the Americas. Recent studies have found a strong association between non-human primate diversity, their mobility patterns through forests, and the presence of human yellow fever cases [327,328]. The importance of howler monkeys (*Alouatta* spp.) as main reservoir host for several arboviruses, such as YFV, and their behavior must be further explored. In Costa Rica, *Alouatta* monkeys spend most of their day (77%) in an inactive state while a smaller proportion of their time is spent moving through the forest, feeding, or engaging in social behavior. In contrast, capuchin monkeys (*Cebus* spp.) spend most of the time (70–80%) day foraging or conducting other active behaviors [329]. The low activity budget of *Alouatta* monkeys could make them more susceptible to mosquito bites and YFV transmission, given that host movement has an important influence on the R0 in vector-borne disease systems [330]. Furthermore, *Alouatta* spp. can adjust their behavior to accommodate different feeding strategies as their forest habitat changes. These monkeys are found in both modified and undisturbed habitats throughout their distribution in Mexico and Central America. Their ability to adapt to changing environments is evidenced in their continued presence in regions where white-faced capuchins and spider monkeys no longer exist [331]. However, recent studies indicate that this behavior may not be spatially consistent. A recent study by Schreirer et al. (2021) in Costa Rica found that *A. palliata* do not appear to adjust their activity or spatial cohesion patterns in response to anthropogenic edge effects due to forest fragmentation, suggesting that these monkeys exhibit less behavioral flexibility than *A. palliata* at some other sites [332]. The high susceptibility of *A. palliata* to YFV infection and movement within disturbed or fragmented habitats could increase the risk of arbovirus transportation and exposure to humans in urbanized forest edges [333,334,335,336].

In Latin America, the relationship between DENV and deforestation has not been clearly established or evidence is scarce. For instance, studies conducted in the Brazilian Amazon have found no association [337,338]. Studies conducted in other endemic DENV regions suggest that land use changes following deforestation (e.g., agriculture, settlements, or road construction) have been identified as significant dengue fever risk factors [300,339]. An increase in human population densities benefits DENV and its vectors through the establishment of artificial breeding habitats (i.e., water storage) and more frequent contact with susceptible populations, thus increasing the risk for virus transmission in rural and urban settings [340]. Other factors, such as proximity to paved roads and house clustering, could also promote breeding habitats [341]. The expansion of urban centers and bordering rural areas closer to the forest edge increases the chance of *Ae. aegypti* dispersal and colonization. For example, a study in the Peruvian Amazon by Guagliardo et al. (2014) found that the geographic spread of *Ae. aegypti* is driven by human transportation networks along rivers and highways in proximity to the city [342]. In this region, urban development and the availability of oviposition sites appear to contribute to the colonization of *Ae. aegypti* along roads. Moreover, unintentional transport of mosquitoes on boats disperses their populations over long distances into rural, riverine communities [342].

Forest fragmentation driven by land use changes could also facilitate movement of adult mosquito vectors between communities. For instance, a mark-release-recapture study by Russell et al. (2005) reported that released *Ae. aegypti* exhibit nonrandom patterns of dispersal with larger proportion of mosquitoes being recaptured along a corridor with heavy shading from trees and vegetation [343]. In Central America, several studies have been conducted using several vegetation indexes to evaluate correlations between vegetation coverage and dengue incidence. For example, a study by Fuller at al. (2009) was able to explain up to 83% of the variability in weekly cases of dengue fever and dengue hemorrhagic fever in Costa Rica between 2003 and 2007 based on vegetation indexes [344]. In another study, high dengue fever incidence correlated spatially with high temperature, low altitude, and a high vegetation index [345]. Moreover, ovitrap egg counts are also associated with vegetation indexes, which are dependent on temperature and rainfall, well known factors affecting vector abundance [346,347,348]. Interestingly, other studies conducted in Central America revealed that dengue cases may be directly associated with tree cover and non-forested areas and inversely associated with built areas, probably because more larval habitats are available in larger, tree-covered outdoor areas with vegetation during rainfall episodes [349,350,351].

In Central America, there are increasing concerns regarding the expansion of *Ae. albopictus* throughout the region. This important arbovirus vector species was initially detected in El Salvador, Honduras, and Guatemala in 1995; later in Panama and Nicaragua in 2002–2003; and Costa Rica and Belize between 2007 and 2009 [352,353,354,355,356]. During the last decade, its distribution in Central America has expanded which is evidenced by surveillance and field study reports [357,358,359,360], as well as by population genetic studies in Panama and Costa Rica [361]. Recent studies in Costa Rica demonstrate the adaptive capacity of this species to changes in land use and expansion of urbanized areas. For example, a study by Calderon-Arguedas et al. (2019) detected all four DENV serotypes in both *Ae. albopictus* female and male adults, and larvae associated with commercial pineapple farming, suggesting local horizontal and vertical transmission of DENV in the region [362]. Moreover, during these studies, most adult *Ae. albopictus* were collected within forest galleries bordering pineapple fields, which may indicate that forest edge habitats could serve as ecological refuges for this species in DENV endemic areas [186,362].

The movement of pathogens from sylvatic to amplified human transmission cycles in rural and urban settings (e.g., disease spillover) have been commonly reported in the literature; however, important questions on specific mechanisms still linger [363]. These events are typically associated with human and animal populations near forest edges, leading occasionally to urban cycles involving peridomestic vectors [304,364]. Arboviral disease spillover events in Central America are possible considering that these viruses share several ecological characteristics, including vectoring by anthropophilic mosquitoes in urban and rural transmission cycles, use of humans as main reservoirs, and their occurrence in habitats associated with expanding agriculture and urbanization near forest edges [150,173,365]. Several studies in Central America suggest the potential for disease spillover of sylvatic arbovirus transmission cycles into rural and urbanized areas. For example, in Panama, evidence suggests that human cases of VEEV infections are associated with spillover infections from an enzootic cycle involving sylvatic rodents and *Cx. (Melanoconion)* spp. mosquitoes [82]. Recent clusters of human cases have occurred in Darien and Panama Provinces near rainforest and swamp habitats. In addition, entry of humans into forest galleries appears to be a risk factor for VEEV transmission [82,113]. Other studies suggest frequent occurrence of spillover events associated with other sylvatic arboviruses in Central America, such as MADV and MAYV [82,118,129,203,211].

Conversely, spillback (e.g., reverse zoonosis) involves the movement of pathogens from urban/rural transmission cycles to sylvatic cycles between wild non-human primates and forest mosquitoes [366]. In the Americas, spillback events have been reported in several countries, including Argentina, Brazil, Colombia, and French Guyana, involving DENV, ZIKV, YFV, CHIKV, neotropical wild primates, and known mosquito vectors [150,363,366,367]. A recent study carried out in Costa Rica found evidence of SLEV, WNV, and DENV seroprevalence in the primate species *A. palliata*, *A. geoffroyi*, and *S. oerstedii* (Reinhardt, 1872). Based on the collection of seropositive samples coinciding temporarily and spatially with peaks of infections in human populations, their study concluded that DENV exposure in these monkeys occurred through bidirectional human–wildlife contact or bridging vectors [85].

The ecological mechanisms by which forest disturbance triggers disease spillback events are still poorly understood, which makes it difficult to predict disease risk scenarios for future outbreaks in human-altered forest habitats and the potential establishment of sylvatic transmission cycles [363,366]. In Central America, where arbovirus-susceptible wild primates and known mosquito vectors are present (e.g., *Haemagogus* spp., *Sabethes* spp.), the occurrence of any one or a combination of epidemiological factors that could drive transmission is highly plausible, considering the high level of deforestation, extensive land use changes, and accelerating development of human settlements near forest edges. It is also possible that immunity of monkeys against active sylvatic arboviruses, such as YFV, could inhibit infections by DENV and ZIKV and, thus, evade the emergence of sylvatic cycles [366]. However, considering that YFV epizootics have not been reported in Central America in decades, cross-immunity to flaviviruses in non-human primates in the region may not be sufficient to suppress other arboviral infections.

Several mosquito species distributed throughout Central America could serve as bridging vectors and initiate spillback events due to their bionomic plasticity. For example, *Ae. albopictus* is found predominantly in urban areas, but also spreads into rural, semi-rural, and forest areas, which could potentially drive arboviruses, such as ZIKV, DENV, and CHIKV, into sylvatic transmission cycles [363,366]. Moreover, sylvatic vectors like *Haemagogus* spp. could also serve as bridging vectors since they can be opportunistic feeders, frequently imbibing on humans and monkeys, in environmentally disturbed regions along forest edges [140,313,321,368]. These mosquito vectors tend to be opportunistic feeders, targeting hosts that are immediately available [308]. This evidence demonstrates that disease spillover is not a random process but may be the result of forest degradation increasing the likelihood of contact between humans and mosquito vectors in forest-altered sites [306,307].

Both spillover and spillback events occur due to increasing human activities near adjacent forest areas and where humans closely interact with wild animals and their pathogens, facilitating the “jump” or “shift” of new pathogens between different host species [302]. Despite our current knowledge of these diseases, it is still difficult to predict the risk for future outbreaks since the ecological mechanisms that drive spillovers and spillbacks in human-modified forest habitats are not well defined. Considering the accelerated rate of deforestation, unplanned urbanization and agricultural expansion near forest edges, high endemicity of multiple arboviruses, and close human contact with wildlife and vectors taking place in Central America, there is a tangible possibility that spillback and spillover events may be more frequent than reported and may surge in the future. Therefore, it is critical that more intense epidemiological and ecological monitoring systems are established in the region to help predict future epizootics and epidemics.

### 5.2. Impact on Malaria

In recent decades, compelling evidence linking deforestation and land use changes with malaria transmission and anopheline vector ecology in Central America have been demonstrated [229,369,370,371]. Deforestation and land use changes can have profound anthropogenic environmental effects on malaria transmission. In general, evidence shows that, in the Americas, deforestation leads to an increased potential for malaria transmission, particularly in communities where the vector *An. darlingi* is present [372,373,374,375,376]. Working specifically in the Darien Province of Panama, Loaiza et al. (2017) correlated increased malaria incidence rates with extensive changes in landscape, including deforestation, and an associated expansion of *An. darlingi* habitat. These findings were recently supported when *An. darlingi* was implicated as an important vector of *P. vivax* in the Darien region [306,377]. Studies in other parts of Latin America have found that the human-biting activity of *An. darlingi* is more intense in areas associated with deforestation and road development [378], while a study by Loaiza et al. (2008) found that the Central American malaria vectors, *An. vestitipennis* and *An. neivai* Howard, Dyar and Knab, 1913 are closely associated with specific forms of vegetation and land-use practices in Panama [217].

Other studies have suggested that agricultural land use changes and environmental pollution can significantly alter natural malaria vector habitats and even affect mosquito diversity driving disease prevalence and more dominant vector populations. For instance, a study by Chapin and Wasserstrom (1981) showed that, as early as the 1970s, expanding the acreage used for cotton cultivation, and the associated increases in pesticide use in Guatemala, Nicaragua, and El Salvador, correlated with both the emergence of DDT resistance in local malaria vectors and rapid increases in annual malaria case incidence rates, in some cases three times greater than previously recorded [379]. Similar findings were noted in Belize, where malathion use in sugarcane cultivation was associated with malathion resistance in local *An. albimanus* populations [380]. Another compelling case study from Belize highlights how phosphorous runoff from sugarcane cultivation in proximity to marshlands increases the amount of dense cattail (*Typha domingensis*) marsh habitat favored by *An. vestitipennis*, the most efficient malaria vector in Northern Belize, and is associated with higher larval densities [381,382]. Interestingly, a strategy to reduce this cattail habitat through environmental management (e.g., mowing and burning) to control *An. vestitipennis* populations in the area was marginally successful, with one important caveat: while the habitat management strategies significantly reduced *An. vestitipennis* larval populations, the resulting altered marshland habitats proved suitable for another important local vector species, *An. albimanus*, whose populations increased [383]. Changes in landscape structure, in the form forest cover percentage and forest density, could also lead to dominant vector populations, such as *An. darlingi*, increasing the risk for malaria transmission [384].

Another aspect of environmental change, like urbanization, is the global expansion of invasive malaria vectors, like *An. stephensi* Liston, 1901, and its potential implications for malaria control and elimination throughout Central America. Often described as a highly competent malaria vector able to breed in human-made containers, *An. stephensi* is known to establish and sustain outbreaks of urban malaria in previously malaria-free regions [385,386,387]. While the arrival and establishment of *An. stephensi* in Central America is probably less likely than in Africa or other parts of Asia [385], it is nonetheless realistic to consider the possibility. Regional receptivity to the vector and its potential to impact local malaria control and elimination initiatives is poorly understood but potentially worrisome given the rapid urbanization of population centers, the high abundance of peridomestic habitats, its invasive potential, and intense human migration from malaria-endemic regions through Central America [388,389].

Although the risk for malaria transmission in Central America has been steadily declining in recent years, the intensification of human migration and illegal drug trafficking throughout the region, which are driving deforestation and land use changes, have the potential to reignite the resurgence of malaria in the region [370]. Currently, the La Mosquitia tropical forest region, located between Honduras and Nicaragua, is one of the most malaria endemic locations in Central America [229]. Although this region is part of the protected MBC, it continues to be threatened by illegal drug smuggling, cattle ranching, logging, and land grabbing [22,38,50]. The recent increase in malaria cases in Panama, Costa Rica, and Nicaragua, especially of the more severe form, *P. falciparum*, raises concerns about the possibility of eradicating malaria in those countries in the next few years. This recent resurgence has been linked to imported cases from foreign migrants passing through the region [390]. While numerous findings highlight the effects of land use and micro and macro habitat changes have on of malaria vectors and disease risk across Central America, it is also evident that more research into these complex relationships and interactions is desperately needed. An improved understanding of anopheline vector ecologies will help inform on how malaria transmission dynamics might change over time and guide future malaria control and elimination activities in the region.

### 5.3. Impact on Tick-Borne Diseases

Studies in different areas of the world have demonstrated that deforestation practices and forest fragmentation could both decrease and modify biodiversity, which may lead to a greater abundance of fewer vertebrate species and an increased prevalence of their associated ticks that can thrive in modified habitats [391,392,393,394,395,396,397]. In addition, some domestic animals, such as dogs and horses, may act as bridges for ticks and zoonotic pathogens from wildlife to humans, resulting in disease spillover events into urban environments [398,399,400,401]. It is also important to consider environmental factors, such as temperature, precipitation and humidity, critical to host and tick survival, which could also change in modified environments. Each host, vector, and pathogen has its own requirements, which must be met to ensure their establishment and survival [394,402,403,404,405,406].

Zoonotic pathogens may resurge depending on the conditions present, such as availability of humans and other domestic animals, tick vectors, and environmental conditions. When conditions are optimal and disease vectors and suitable vertebrate reservoir hosts become abundant, pathogen transmission risk may increase. Decreased contact between ticks and vertebrate hosts not relevant in pathogen transmission can also increase disease risk, which could cause a dilution effect. However, the mechanisms of this effect depend on scale and overall context, including local land use changes, climate/microclimate conditions, host communities, and their interactions [395,397]. Therefore, if humans encounter tick vectors in these deforested or disturbed areas, there may be a greater risk of zoonotic disease transmission.

In general, the scientific literature concerning tick-borne diseases in Central America focuses on the distribution or presence of tick species and their associations with vertebrate hosts and reports of human infections by specific pathogens (for examples see [233,234,236,238,241,243,244,247,249,253,254,255,257,259,261,262,263,264,278,285,293,404,407,408,409,410,411,412,413]). However, very few studies in the region have focused on the influence of land use changes on pathogen and vector ecology. Except for recent studies in Panama [405,414,415], no other published reports in the scientific literature have directly and specifically investigated the effects of forest modification, deforestation, or land use changes on tick species, or on pathogen transmission dynamics.

Among the few studies conducted in the region, one conducted in the Chiriquí province of Panama aimed to identify environmental predictors of tick burdens on dogs, as well as environmental predictors of pathogens in these ticks, including vegetation cover and land use change [405]. Although the most relevant predictor of tick prevalence and abundance was elevation, a decrease in vegetation cover linked to increased urbanization, was also associated with the highest tick prevalence and abundance. This is probably due to the close relationship of *R. sanguineus* s.l., the most common dog tick in the region, with human dwellings [405]. In areas with higher vegetation cover and less urbanization, other tick species became relevant, including *A. ovale* and *I.* c.f. *boliviensis*. Therefore, deforestation linked to increased urbanization appears to hinder survival and establishment of ticks, such as *A. ovale* and *I.* cf. *boliviensis*, while benefitting *R. sanguineus* s.l. [405]. In areas where *R. sanguineus* s.l. may act as a vector of zoonotic pathogens, decreases in forest cover and urbanization may increase transmission risk to humans.

Another study in Panama investigated tick diversity in a gradient of decreasing disturbance (low trees and shrubs, secondary forest, secondary forest crossed by a creek, and secondary–primary transition forests also crossed by a creek) and increasing forest cover along the 17 km Oleoducto trail in Soberania National Park [414]. Results showed that the most disturbed site had fewer tick–host interactions, compared with the other sites, and showed low tick diversity and few potential hosts [414]. Notably, this site included more *A. mixtum* ticks, which are common in diverse environments, including disturbed landscapes. Moreover, secondary and transition forests had a higher diversity of tick species and tick-host interactions, including a high abundance and diversity of birds and small mammals as well as several medium and large sized mammals that could serve as hosts for different tick species [414]. Therefore, this study suggests that a decrease in tick and host diversity may be a consequence of deforestation and forest disturbances in Central America. If these tick populations and their small mammal hosts are relevant in zoonotic transmission, deforestation in the region may also increase the risk for human infections due to diversity loss [395,397].

Additionally in Panama, a recent study investigated both vertebrate and tick communities in forest fragments, specifically in forested islands and peninsulas in the Barro Colorado Nature Monument, which were formed as a result of of damming the Chagres River about a century ago [415]. Its main findings indicate that tick species richness and abundance in this area increases according to the availability of vertebrate host species richness and wildlife biomass, which is higher in larger forest patches. In addition, tick species that have a broad range of vertebrate hosts as adults (e.g., generalists) increase in abundance when host diversity and specialist tick species is low [415]. Therefore, when human activities cause forest fragmentation, there is a decrease in wildlife biodiversity, while smaller sized tick hosts and generalist tick species may become more abundant. When these include possible reservoirs and vectors of pathogens, the risk of transmission between vertebrate species, including humans, can also increase [397].

Other studies in Panama have documented that most local species of ticks are host- specific as adults or at least they are associated with taxonomically related vertebrate species [416]. Therefore, a notable change in host diversity driven by deforestation and other land use changes could result in significant changes in local tick populations and disease transmission dynamics. For instance, the most common ticks on bovines in Central America are *Rhipicephalus microplus* (Canestrini, 1888) and *Amblyomma mixtum* (formerly cited as *Boophilus microplus* and *A. cajennense*, respectively), whereas horses are usually parasitized by *Dermacentor nitens* Neumann, 1897 (=*Anocentor nitens*) and *A. mixtum* [231,234,259,402,404,408,417,418,419]. Depending on the specific area, other frequently found species include *A. maculatum* Koch, 1844, *A.* cf. *oblongoguttatum* Koch, 1844, and/or *A. parvum* Aragão, 1908, among others [408,418,419]. Therefore, deforestation associated with cattle ranching may increase local populations of these ticks driven by changes in host abundance if other environmental conditions are suitable for ticks to complete their development. The case of *A. mixtum* is of particular interest since this species is considered a generalist biter frequently feeding on humans in areas of Central America [276,297,404,407,410,411,420]. This tick species has been identified as a vector of *R. rickettsii* in Costa Rica and Panama [237,404,411,421]. Moreover, there is molecular evidence of other pathogens detected in *A. mixtum* in Central America, including *E. chaffeensis* [237]. *Amblyomma maculatum* is the main vector of *R. parkeri*, which has been reported in Belize [413,422]. It is also relevant to note that there are additional reports of bovines and equines infected by *A. phagocytophilum* and detections of *E. chaffeensis*, *E. ewingii*, and/or *A. phagocyophilum* DNA in unidentified *Amblyomma* sp., *D. nitens*, and *R. microplus* collected from horses and cows in Guatemala and Panama [234,236]. Furthermore, *R. microplus* and *D. nitens* can also parasitize white tail deer (*Odocoileus virginianus* [Zimmermann, 1780]) in areas where they coexist with cattle and horses [407,423,424]. In countries like Brazil, infection of *R. rickettsii* in humans has been associated with a shift from predominantly rural to a more urban transmission in Rio de Janeiro where ticks like *D. nitens*, *R. microplus*, and *A. sculptum* Berlese, 1888 are common on horses and cattle [398]. In this area, *A. sculptum* is also common on wild animals and it is considered a generalist biter and the most important vector of *R. rickettsii*. In Central America, an increase in the abundance of cattle, horses, and *A. mixtum* (and other generalist ticks) in areas where there is already local transmission of zoonotic pathogens may result in an increased risk of human exposure to these potential vectors and pathogen infection.

*Rhipicephalus sanguineus* s.l. is the most common tick on domestic dogs in Central America, although it is not indigenous to this region [241,249,404,408,425,426,427]. Despite its marked preference toward dogs, this tick also bites humans and can do so frequently in rural and urban areas in Central America [297,420]. Considering that these ticks are usually found in urban environments or areas that have been disturbed, deforestation and other land use changes leading to human settlements and increased dog populations could result in the establishment of this species where it was not present before human activity, as it has been observed in Panama [405]. In addition to being the main vector of *E. canis* in the region, *R. sanguineus* s.l. may be implicated in the transmission of *A. phagocytophilum*, given reports of infection in dogs in Costa Rica and Nicaragua and the detection of its DNA in this tick species in Costa Rica and Panama [239,249,255,257,404]. Moreover, *R. sanguineus* s.l. has been identified as responsible for urban outbreaks of severe human rickettsiosis by *R. rickettsii* in Mexico, and it may have been associated with a human case in Panama [277,428]. Considering the presence of these bacteria in Central America, an increase in dog populations in this region may facilitate contact with pathogen transmission cycles in wildlife and their ticks. In Rio de Janeiro, Brazil, recent investigations close to the Pedra Branca State Park have found *R. rickettsii* in *R. sanguineus* ticks as well as a higher exposure of dogs to *Rickettsia* spp. in recently urbanized areas, compared to rural and non-endemic areas [429]. This supports the hypothesis that interactions of dogs with wildlife and their ticks may eventually lead to the establishment of urban transmission cycles among dogs and *R. sanguineus* s.l. vectors and the possibility of a higher risk to humans mediated by contact with *R. sanguineus* s.l.

*Amblyomma ovale* is another tick species in Central America that can be found feeding on dogs in rural areas, urban periphery, and nearby human-disturbed forests areas or frequently accessed forest habitat (e.g., for hunting) [241,254,404,407,408,411,425,426,430,431]. Although it is not frequently found in highly urbanized areas, this species readily bites humans in rural landscapes of Central America and it is the main vector of *R. parkeri* (strain Atlantic rainforest) in other parts of Latin America, including Colombia, Brazil, and Argentina [297,404,420,422,431,432,433,434]. The presence of this strain of *R. parkeri* and *R. africae* in *A. ovale* has been recently reported in Belize and Nicaragua, respectively [254,412]. Moreover, several studies in the Americas, such as the U.S. and Brazil, have found that people who own or hunt with dogs that are in contact with wildlife and forest areas are more exposed to wildlife ticks, including vectors of zoonotic pathogens, that could also parasitize dogs [399,400]. Therefore, a consequence of increased human settlements in proximity to forest habitats and incursions into nearby forests, along with their domestic dogs for company, herding, hunting, and other activities, may increase human and dog exposure to *A. ovale* and other ticks and wildlife pathogens in disturbed and forest areas of Central America.

Land use changes leading to human settlements may also increase contact of humans with argasid ticks and the pathogens that they carry. Tick infestations in domestic environments and human bites by *Ornithodoros* spp. in Central America can occur due to bats, rodents, birds, or other small animals accessing the interior of buildings or seeking refuge close to buildings [248,293,297,435,436,437]. In Panama, *O. talaje* and *O. rudis* were implicated as vectors of an unidentified *Borrelia* sp. that causes tick-borne relapsing fever in the area, but other common species (e.g., *O. puertoricensis* [Fox, 1947]) may also be competent vectors of *Borrelia* spp. [248,437]. In other regions of the world where cases of tick-borne relapsing fever occur, infection usually takes place within houses or buildings infested with argasid ticks [248,438]. In Central America, the expansion of human settlements, especially informal and unplanned housing, could offer adequate refuge for argasid ticks and small animals, such as rodents and bats, placing humans in close proximity to these ticks and increasing the risk for pathogen transmission.

There are other potential tick-borne pathogens in Central America that have been directly or indirectly documented in wild animals or ticks, but not in humans. For example, DNA of *A. phagocytophilum* and *B. burgdorferi* s.l. was recently detected in *I. tapirus* Kohls, 1956 and in *I.* cf. *boliviensis*, respectively, in Panama [263]. Although there is evidence of possible exposure to *B. burgdorferi* s.l. in dogs and humans in Central America, the bacterium involved has not been clearly identified [257,265,270,439]. However, species such as *I.* c.f. *boliviensis* have the potential to transmit enzootic pathogens and may parasitize domestic animals, including dogs and even humans [255,297,407,425,426,440]. This generalist feeding behavior may allow them to act as bridge vectors in peridomestic environments, especially near disturbed forests and rural settings near forests. The transmission cycles of *A. phagocytophilum* and *B. burgdorferi* s.s. typically involve wild animals, such as white tail deer and rodents, and tick populations that may increase in abundance due to forest disturbances, conservation measures, and reforestation practices [391,393,396,441,442].

## 6. Conclusions

The recent epidemic emergence of several VBDs in Central America highlight the importance of elucidating the specific regional factors that drive their emergence, which could lead to the development of strategies to prevent their further spread and establishment. In the last several decades, climate change has received major attention and it is considered an important driver of VBD emergence and resurgence in Central America, especially for malaria and DENV [443,444,445,446]. However, less attention has focused on the influence of land use changes on VBD emergence and resurgence in this region. Understanding environmental drivers, such as urbanization and deforestation, is critical since these factors could far exceed the rate of climate change and have been directly linked to the spread of several VBDs in the neotropics [16,447,448,449,450]. Furthermore, the Central American region is currently experiencing one of the fastest levels of deforestation and urbanization in the world, which are considered amongst the most important drivers of VBD emergence [16]. The increased movement of people to and from forests also promotes and facilitates contact between insect vectors, reservoirs, and human hosts. These interactions may serve as bridges for pathogens to reach human populations beyond forest edges into urbanized regions [5]. The development of natural resources can lead to habitat simplification and reduction in biodiversity which may also affect the ecology of local disease vectors. Moreover, excessive use of pesticides could also lead to the accelerated development of insecticide resistance amongst disease vectors [1].

Another factor impacting VBD transmission dynamics is the increase in intensity and number of natural disasters due to climate change [5]. As tropical storms and hurricanes are becoming more common and severe, disease vectors have an increased potential to disperse to areas in which new breeding sites may be more suitable for their establishment [451,452]. As a result of global increases in urbanization and temperatures, areas suitable for breeding and proliferation of disease vectors may expand which, in turn, may increase the risk for pathogen transmission to humans and animals [5,453].

Knowledge gaps on the epidemiology and ecology of VBDs in Central America are not geographically uniform. As reported in this review, most studies on this subject have been conducted in Panama and Costa Rica, countries with the strongest economies and public health infrastructures in the region [30]. Over the past few decades, Central America has encountered considerable social and environmental challenges linked to climate change, including extended droughts combined with intermittent and extreme floods. Moreover, the region continues to experience political instability and violence due to the illegal drug trade, socioeconomic instability, food insecurity due to agricultural declines, substantial human displacements, unplanned urbanization, and the marginalization of Indigenous populations [454,455]. Moreover, marked differences between countries exist, including various levels of public health infrastructure development, disease surveillance capabilities, and availability of effective laboratory testing technology [6].

This review set out to explore the potential impact of accelerating rates of deforestation, urbanization, and other anthropogenic changes on VBD transmission dynamics in Central America, focusing on mosquito-borne and tick-borne diseases with high potential to emerge, expand, or resurge. Throughout the region, changes in current land use practices are influencing the unique ecological, social, and environmental determinants of health in ways that are interdependent, synergistic, and difficult to study. As a result, it is likely that a growing number of people in Central America are at increased risk for VBDs. However, the specific effects of environmental changes, such as deforestation and urbanization, on VBD transmission dynamics are not yet well understood and are challenging to predict, which highlights the critical need for increased surveillance and further study in the region. A more detailed understanding of the complex relationships between the unique VBD ecologies and rapidly changing environments in Central America is urgently needed to inform rational disease prevention and control activities across the region.

## Figures and Tables

**Table 1:** Current proportions of forest area, agricultural land, and urban population in Central America, including changes over the last 30 years.

Country	^a^ Total Country Area (sq. km.)	^b^ Forest Area, % of Land Area (2018)	^c^ Change in Forest Area, % of Land Area (1990–2018)	^d^ Agricultural Land, % of Land Area (2018)	^e^ Change in Agricultural Land, % of Land Area (1990–2018)	^f^ Urban Population % (2020)	^g^ Change in Urban Population % (1990–2020)
Belize	22,810	57	−13.2	7.5	+2	46	−1.44
Costa Rica	51,060	58.8	+1.9	34.9	−8.1	80.8	+30.8
El Salvador	20,720	28.6	−6.1	71.4	+6.2	73.4	+24.2
Guatemala	107,160	33.1	−11.5	36	−4	51.8	+9.8
Honduras	111,890	57.2	−5.2	30	+0.3	58.4	+17.9
Nicaragua	120,340	30	−23.2	42.1	+8.6	59	+5.9
Panama	74,177	57.1	−4.9	30.5	+1.9	68.4	+14.5

^a^ Data source: The World Bank https://data.worldbank.org/indicator/AG.LND.TOTL.K2?view=map (accessed on 18 August 2021). ^b^ Data source: The World Bank https://data.worldbank.org/indicator/AG.LND.FRST.ZS?view=map (accessed on 18 August 2021). ^c^ Data source: The World Bank. Value calculated by determining the difference in forest area (% of land) between 1990 and 2018 per country https://data.worldbank.org/indicator/AG.LND.FRST.ZS?view=map (accessed on 18 August 2021). ^d^ Data source: The World Bank https://data.worldbank.org/indicator/AG.LND.AGRI.ZS?view=map (accessed on 18 August 2021). ^e^ Data source: The World Bank. Value calculated by determining the difference in agricultural land area (% of land) between 1990 and 2018 per country https://data.worldbank.org/indicator/AG.LND.AGRI.ZS?view=map (accessed on 18 August 2021). ^f^ Data source: The World Bank https://data.worldbank.org/indicator/SP.URB.TOTL.IN.ZS?view=map (accessed on 18 August 2021). ^g^ Data source: The World Bank. https://data.worldbank.org/indicator/SP.URB.TOTL.IN.ZS?view=map. Value calculated by determining the difference in urban population % between 1990 and 2020 per country (accessed on 18 August 2021).

**Table 2:** Description of the most important mosquito-borne and tick-borne diseases in Central America.

Disease	Causative Agents	Distribution of Infections in Humans	Confirmed or Suspected Mosquitoes and/or Tick Vectors	Confirmed or Suspected Non-Human Vertebrate Hosts
West Nile fever	West Nile virus (*Flavivirus*)	Clinical, serosurveys (CR, N)	*Culex quinquefasciatus*, *Cx. mollis/Cx. inflictus* (G)	Equines, non-human primates, wild birds, sentinel chickens (CR, B, ES, G)
Saint Louis encephalitis	Saint Louis encephalitis virus (*Flavivirus*)	Clinical, serosurveys (P, B, G, H)	*Sabethes chloropterus, Trichoposopon* spp., *Wyeomyia* spp., *Haemagogus lucifer*, *Deinocerites pseudes*, *Mansonia dyari, Culex nigripalpus* (P, CR, G)	Wild rodents, wild birds, sentinel rodents, sentinel chickens, non-human primates, sloths, equines, pigs (P, CR, B, H, G)
Venezuelan equine encephalitis	Venezuelan equine encephalitis virus (*Alphavirus*)	Clinical, serosurveys (all countries)	Psorophora confinnis, Culex nigripalpus, * Cx. (Melanoconion) taeniopus, other Cx. (Melanoconion) spp., *Mansonia titillans, Ps. cilipes, Aedes taeniorhynchus*, and *Deinocerites pseudes* (P, CR, B, G)	Equines, wild rodent, opossum, birds, and bats (CR, N, H, ES, G)
Eastern equine encephalitis	Madariaga virus (*Alphavirus*)	Clinical, serosurveys (P)	*Culex (Mel.) taeniopus* (P)	Horses, bats, wild lizards, wild birds (P, CR, B)
Yellow fever	Yellow fever virus (*Flavivirus*)	Clinical (all countries)	*Aedes aegypti, Haemagogus janthinomys, Hg. leucocelaenus, Hg. lucifer*, *Hg. equinus, Hg. spegazzinii*, and *Sa. chloropterus, Hg. mesodentatus* (P, CR, N, G)	Non-human primates, marsupials (P, CR, N, B, H, G)
Zika fever	Zika virus (*Flavivirus*)	Clinical and serological (all countries)	** *Aedes aegypti, Ae. albopictus* (all countries)	Unknown
Chikungunya fever	Chikungunya virus (*Alphavirus*)	Clinical and serological (all countries)	** *Aedes aegypti, Ae. albopictus* (all countries)	Unknown
Dengue fever	Dengue viruses 1–4 (*Flavivirus*)	Clinical and serological (all countries)	** *Aedes aegypti, Ae. albopictus* (suspected) (all countries)	Bats, non-human primates (CR)
Mayaro fever	*Mayaro virus (Alphavirus)*	Clinical and serological (P, CR, G)	*Haemagogus janthinomys*, *Psorophora ferox*, *Culex* (*Mel.*) *vomerifer* (P)	Non-human primates (P, CR, H, G)
Malaria	*Plasmodium vivax*, *P. falciparum*	Clinical and serological (all countries)	* *Anopheles albimanus, An. darlingi, An. punctimacula*, other *Anopheles* spp. (all countries)	Unknown
	*P. malariae*	Clinical and serological (P, CR, B, ES, G)	Unknown	Unknown
Rickettsiosis	*Rickettsia* spp. (species causing spotted fevers was not identified)	Clinical, serosurveys (all countries)	*Amblyomma mixtum* (G)	Wild rabbits, dogs, coyote (P, CR)
	*R. rickettsii* (Rocky Mountain spotted fever)	Clinical (P, CR)	* *A. mixtum, Rhipicephalus sanguineus* s.l., *A. varium*, *Dermacentor nitens, Haemaphysalis leporispalustris* (P, CR)	Dog, horse (P, CR)
	*R. akari* (rickettsialpox)	Serosurvey (CR)	Unknown	Unknown
	*R. parkeri*	Unknown	* *A. maculatum* (B)	Unknown
	*R. parkeri* strain Atlantic Forest	Unknown	* *A. ovale* (B)	Unknown
	*R. africae*	Unknown	*A. ovale* (N)	Unknown
Ehrlichiosis	*Ehrlichia chaffeensis* (monocytic ehrlichiosis)	Clinical (CR)	*Amblyoma mixtum*, *Amblyomma* sp., *Dermacentor nitens*, *Rhipicephalus microplus* (P)	Unknown
	*E. ewingii* (monocytic ehrlichiosis)	Unknown	*R. microplus* (P)	Unknown
	*E. canis* (granulocytic ehrlichiosis)	Clinical (P, CR)	** *R. sanguineus* s.l. (all countries)	Dogs (all countries)
Anaplasmosis	*Anaplasma phagocytophilum* (human granulocytic anaplasmosis)	Unknown	*Rhipicephalus sanguineus* s.l., *R. microplus* (P, CR)	Dogs, bovines, equines, deer (CR, N, G)
Borreliosis	*Borrelia burgdorferi* s.l. (Lyme disease)	Clinical (CR)	*Ixodes* c.f. *boliviensis* (P)	Dogs (CR)
	*Borrelia* sp. (tick-borne relapsing fever)	Clinical (P, G)	*Ornithodoros talaje, O. rudis* (P)	Armadillos, opossums (P)

* Main vector. ** Suspected vectors; pathogen has not been detected/isolated in all countries from these vectors, but they are present and considered the main vectors worldwide. Country abbreviation (Belize = B; Costa Rica = CR; El Salvador = ES; Guatemala = G; Honduras = H; Nicaragua = N; Panama =P).

## Data Availability

This review article does not report original data results; hence, the statement is excluded.
